# Purine Metabolism and Dystonia: Perspectives of a Long‐Promised Relationship

**DOI:** 10.1002/ana.27227

**Published:** 2025-03-03

**Authors:** Ugo Sorrentino, Audrey G. O'Neill, Justin M. Kollman, Hyder A. Jinnah, Michael Zech

**Affiliations:** ^1^ Institute of Human Genetics, Technical University of Munich, School of Medicine and Health Munich Germany; ^2^ Department of Biochemistry University of Washington Seattle WA; ^3^ Departments of Neurology, Human Genetics and Pediatrics Emory University School of Medicine Atlanta GA; ^4^ Institute of Neurogenomics, Helmholtz Munich Neuherberg Germany; ^5^ Institute for Advanced Study, Technical University of Munich Garching Germany

## Abstract

Dystonia research focuses on the identification of converging biological pathways, allowing to define molecular drivers that serve as treatment targets. We summarize evidence supporting the concept that aberrations in purine metabolism intersect with dystonia pathogenesis. The recent discovery of *IMPDH2*‐related dystonia introduced a gain‐of‐function paradigm in purinergic system defects, offering new perspectives to understand purine‐pool imbalances in brain diseases. We discuss commonalities between known dystonia‐linked mechanisms and mechanisms emerging from studies of purine metabolism disorders including Lesch–Nyhan disease. Together, we hypothesize that a greater appreciation of the relevance of purine perturbances in dystonia can offer fresh avenues for therapeutic intervention. ANN NEUROL 2025;97:809–825

## Graphical abstract

Purine pool imbalance in purine metabolism disorders, such as Lesch–Nyhan disease, intersects with dystonia pathogenesis. The recent discovery of gain‐of‐function *IMPDH2* pathogenic variants in patients with prominent dystonic manifestations provides new insights into the link between dystonia and purinergic system defects. New perspectives on brain purine‐pool imbalances and pathophysiology of dystonia may offer fresh avenues for therapeutic intervention. Created with Biorender.com.

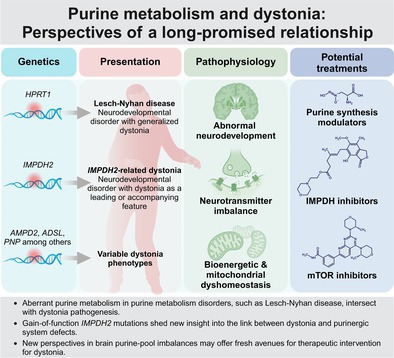

Adenine and guanine, together with their derivatives, comprise a group of ubiquitous aromatic heterocyclic molecules collectively referred to as purines. In their base form or as part of more complex biomolecules, purines are essential constituents of all living cells.^1^ Although it is well established that defects of purine metabolism underlie diverse clinical disorders,^2^, ^3^, ^4^ an important underexplored question is to what extent abnormalities of purinergic system function may cause dystonia and whether this could involve intersections with other molecular mechanisms associated with dystonia. Insights into the relationship between dystonia and pathological deregulation of purine pathways may offer unique opportunities to understand dystonia pathogenesis and identify new targets for treatment.

Purines are involved in diverse biological processes, including the formation of nucleic acids (DNA, RNA), bioenergetic substrates (adenosine triphosphate [ATP], coenzyme‐A, nicotinamide adenine dinucleotide + hydrogen [NADH], nicotinamide adenine dinucleotide phosphate [NADPH], Flavin adenine dinucleotide [FADH2]), as well as intracellular (cyclic guanosine monophosphate [cGMP], cyclic adenosine monophosphate [cAMP]) and extracellular (ATP, adenosine diphosphate [ADP], adenosine, inosine) signaling molecules.^5^ As a consequence, purine metabolism is indispensable for organismal development and survival. In humans, purines can be directly assimilated through dietary ingestion, synthesized de novo from substrates of the pentose phosphate pathway, or recycled by salvaging preformed purine bases (adenine, guanine, and hypoxanthine).^6^ Purines that are not salvaged are ultimately catabolized to uric acid.^7^ Both de novo synthesis and salvaging are accomplished by a complex interconnected network of enzymes and coenzymes.^8^, ^9^, ^10^ The salvage pathway represents the baseline route for regenerating and maintaining the turnover of the purine pool, whereas de novo synthesis is more active under conditions of high purine demand, such as tissue growth and repair.^11^ However, their function is complementary and tightly regulated by substrate availability, cellular requirements, and tissue‐specific influences.^12^ The central nervous system (CNS), where molecules such as ATP and adenosine play pivotal roles as energetic substrates, neurotransmitters, and neuromodulators, strongly depends on fine‐tuned purinergic system function.^13^, ^14^

Considering the direct or indirect involvement of purines in various aspects of human physiology, it is unsurprising that genetic variants disrupting purine‐metabolism genes have been linked to a growing spectrum of monogenic disorders (purine metabolism disorders [PMDs]) (Fig 1).^2^, ^15^ Genetically determined abnormalities of purine metabolism predominantly follow autosomal recessive and X‐linked modes of inheritance, consistent with a loss of enzyme function (see Fig 1). Although the involved enzymes are implicated in the same metabolic pathway, the variability of observed clinical outcomes is remarkable.^4^ The associated phenotypes range from isolated or syndromic immune‐system deficiencies to multisymptomatic developmental disorders. In most cases, the precise pathogenic mechanisms connecting the specific enzymatic defect to the phenotype have not yet been fully characterized.

**FIGURE 1:**
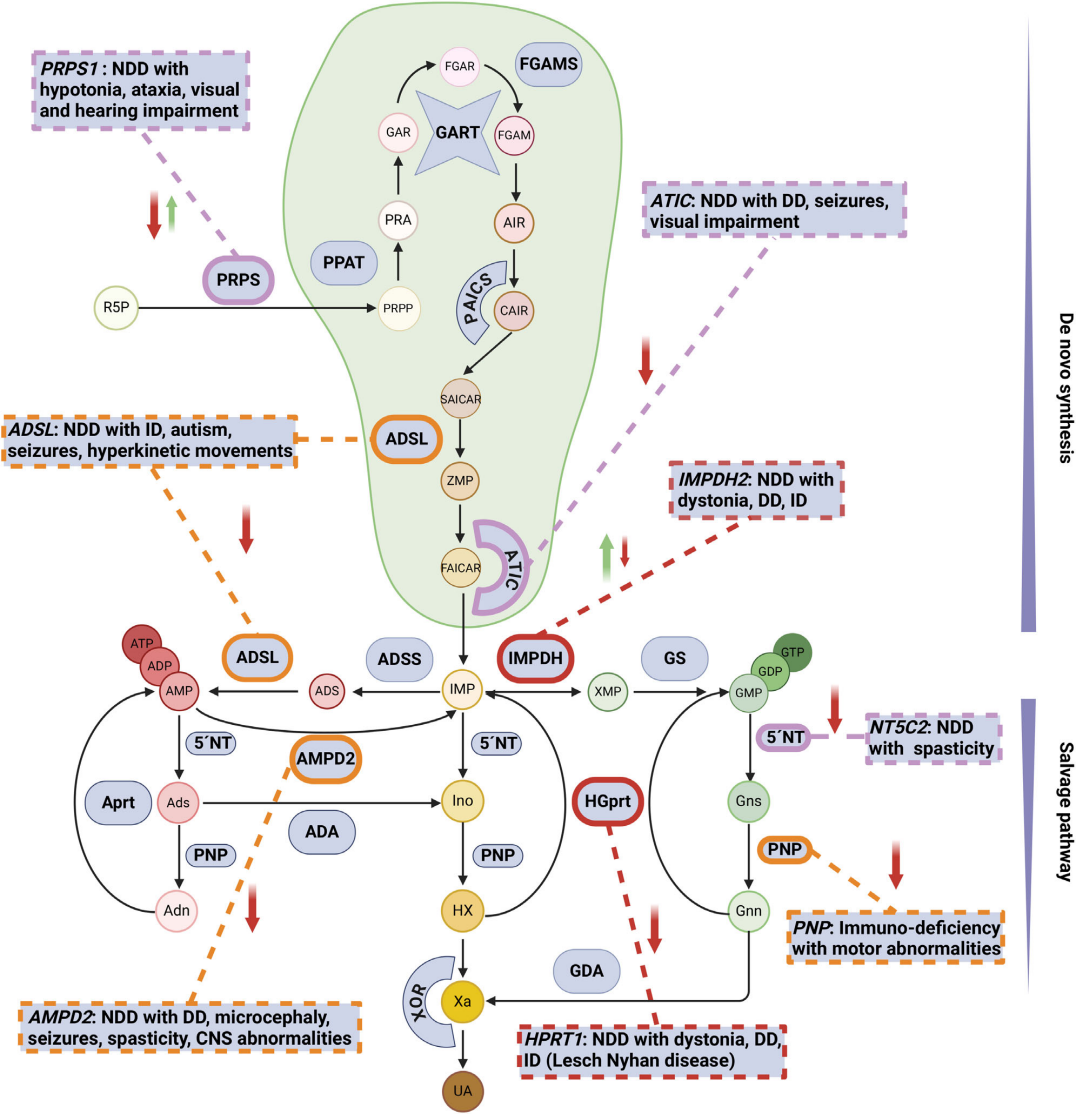
Schematic of the purine de novo synthesis and salvage pathway and associated monogenic neurodevelopmental disorders. Maintenance of physiological purine levels is the result of 2 interconnected metabolic processes, the de novo synthesis and the salvage pathway. In situations of increased purine demands, ribose 5‐phosphate (R5P), a product of the pentose phosphate pathway, is anabolized to phosphoribosyl pyrophosphate (PRPP). Subsequently, it undergoes a series of reactions catalyzed by an assembly of 6 enzymes, collectively known as the purinosome (green balloon), and is ultimately transformed into inosine monophosphate (IMP). Depending on metabolic requirements, IMP can be directed toward the synthesis of guanine nucleotides, through a series of reactions initiated by inosine monophosphate dehydrogenase (IMPDH), or toward adenine nucleotides, by means of the tandem reactions mediated by the enzymes adenylosuccinate synthase (ADSS) and adenylosuccinate lyase (ADSL). As soon as purine nucleotides are expended as a consequence of their physiological functions, they are further catabolized by the enzymes 5′‐nucleotidase and purine nucleoside phosphorylase and ultimately excreted as uric acid. To avoid wasting of difficult‐to‐replace substrates, a salvaging system exists, enforced by the 2 enzymes adenine phosphoribosyltransferase (Aprt) and hypoxanthine‐guanine phosphoribosyltransferase (HGprt), which is capable of restoring the purine pool without resorting to the energetically demanding de novo synthesis. Enzymes from both pathways have been associated with neurodevelopmental disorders, whose main clinical features are summarized in the rectangular boxes. Disorders caused by HGprt and IMPDH disruptions (bold red) have dystonia as a main phenotypic feature. Disorders linked to ADSL, AMPD2, or PNP deficiency (bold orange) have also been associated with dystonia in some cases. Gain‐of‐function and loss‐of‐function pathogenic mechanisms for each enzyme are indicated with a green upward arrow and a red downward arrow, respectively. 5′NT = 5′‐nucleotidase; ADA = adenosine deaminase; Adn = adenine; ADP = adenosine diphosphate; Ads = adenosine; ADS = adenylosuccinate; ADSL = adenylosuccinate lyase; ADSS = adenylosuccinate synthase; AIR = aminoimidazole ribonucleotide; AMP = adenosine monophosphate; AMPD2 = adenosine monophosphate deaminase 2; Aprt = adenine phosphoribosyltransferase; ATIC = aminoimidazole carboxamide ribonucleotide formyl transferase/IMP cyclohydrolase; ATP = adenosine triphosphate; CAIR = carboxyaminoimidazole ribonucleotide; DD = developmental delay; FAICAR = formyloaminoimidazole carboxamide ribonucleotide; FGAM = formylglycinamidine ribonucleotide; FGAMS = formylglycinamidine ribonucleotide synthase; FGAR = formylglycinamide ribonucleotide; GAR = glycinamide ribonucleotide; GART = glycinamide ribonucleotide transferase; GDA = guanine deaminase; GDP = guanosine diphosphate; GMP = guanosine monophosphate; Gnn = guanine; Gns = guanosine; GS = guanosine monophosphate synthetase; GTP = guanosine triphosphate; HGprt = hypoxanthine‐guanine phosphoribosyltransferase; HX = hypoxanthine; ID = intellectual disability; IMP = inosine monophosphate; IMPDH = inosine monophosphate dehydrogenase; Ino = inosine; NDD = neurodevelopmental disorder; PAICS = phosphoribosyl aminoimidazole carboxylase/ phosphoribosyl aminoimidazole succinocarboxamide synthetase; PNP = purine nucleoside phosphorylase; PPAT = phosphoribosylpyrophosphate amidotransferase; PRA = phosphoribosylamine; PRPP = phosphoribosyl pyorphosphate; PRPS = phosphoribosylpyrophosphate synthetase; R5P = ribose 5‐phosphate; SAICAR = succinylaminoimidazole carboxamide ribonucleotide; UA = uric acid; Xa = xanthine; XMP = xanthine monophosphate; XOR = xanthine oxidoreductase; ZMP = Z‐nucleotide monophosphate. The figure was created with Biorender.com. [Color figure can be viewed at www.annalsofneurology.org]

Despite being understudied, early efforts have been made toward evaluating the relevance of dystonia among the phenotypic manifestations of PMDs.^16^ Lesch–Nyhan disease (LND), which is caused by the lack of activity of hypoxanthine‐guanine phosphoribosyltransferase (HGprt), has been most consistently associated with dystonia.^16^ More rarely, dystonia has been reported as a comorbid clinical feature in syndromic patients with pathogenic variants in adenosine monophosphate deaminase 2 (AMPD2),^17^ adenylosuccinate lyase (ADSL),^18^ and purine nucleoside phosphorylase (PNP).^19^ Nevertheless, it remains unclear whether dystonia should be considered a more general trait related to perturbations of purine metabolism. Only recently, a growing body of evidence highlighted a causal relationship between both gain‐ and loss‐of‐function variants in 1 key enzyme of the purine de novo synthetic pathway, inosine‐5‐prime‐monophosphate dehydrogenase type 2 (IMPDH2), and dystonia.^20^, ^21^, ^22^

In this review, we discuss how knowledge of the role of dystonic features in PMDs is expanding, especially because of the discovery of IMPDH2‐related dystonia, and how the new observations can help to redefine the role of purine metabolism aberrations in dystonia etiology. We outline pathophysiological concepts of purinergic system dysfunction with regard to CNS disease states, with a view on emerging links to dystonia‐relevant molecular pathways. Finally, we highlight how a deeper understanding of this topic could pave the way for novel therapeutic avenues in the field of dystonia.

## The Old Guard: LND

LND is the most comprehensively studied disorder linking purine metabolism alterations to dystonia.^23^ The condition is caused by X‐linked hemizygous variants in *HPRT1* encoding HGprt. By catalyzing the transfer of a 5‐phosphoribosyl group from 5‐phosphoribosyl 1‐pyrophosphate (PRPP), the enzyme is responsible for salvage of the purines hypoxanthine and guanine, anabolizing them to inosine monophosphate (IMP) and guanosine monophosphate (GMP), respectively (see Fig 1). Reduced amounts of HGprt activity lead to accumulation of its substrates hypoxanthine and guanine, as well as the cosubstrate PRPP.^24^, ^25^ The unsalvageable products escape the pathway, causing depletion of cellular purine reserves, and are ultimately degraded to uric acid, whereas excess PRPP stimulates purine de novo synthesis, exacerbating metabolic decompensation.^26^ A discussion of the structure of HGprt and a more detailed description of the biochemical consequences of defective HGprt are beyond the remit of this review.^27^

Individuals with LND exhibit a spectrum of neurological and neuropsychiatric abnormalities, including developmental delay, dystonia, choreoathetosis, ballism, spasticity, intellectual disability, and recurrent self‐injurious behaviour.^16^ Dystonia in LND is often generalized in distribution and aggravated by action with superimposition on a baseline of hypotonia. In many patients, it represents the most disabling motor feature. In a multicenter evaluation of 44 individuals with LND, dystonia usually showed onset during the first 2 years of life and manifested as multidirectional cervical dystonia, blepharospasm, oromandibular dystonia, opisthotonus, and/or limb dystonic movements with fixed distal postures. This motor disorder closely resembles dyskinetic cerebral palsy.^16^ Seizures and systemic anomalies such as testicular atrophy and anemia have also been reported in a relevant proportion of cases. Over‐production of uric acid is a universal characteristic of the condition.^28^

To date, more than 600 different pathogenic *HPRT1* variants have been described in families with LND‐affected individuals.^29^, ^30^ Approximately 80% of identified variants are loss‐of‐function single‐nucleotide variants or small insertion–deletion changes, including nonsense, frameshift, splice‐site, and missense mutations, often resulting in complete or near‐complete absence (<1.5%) of HGprt enzymatic activity. The remaining 20% of HGprt deficiency‐inducing sequence alterations are structural defects, mainly single‐ or multi‐exon deletion copy‐number variations.^31^ A comprehensive overview of LND‐associated *HPRT1* variants has been provided previously.^29^, ^32^ LND affects almost exclusively male patients, with transmission of the causative variants from heterozygous non‐manifesting carrier mothers. Occasionally, manifesting females have been observed in the context of non‐random X‐inactivation.^33^

## The New Upstart: *IMPDH2* ‐Related Dystonia

In 2020, a large‐scale genomic study that integrated global matchmaking for rare‐disease cases reported on the discovery of autosomal dominant heterozygous variants in IMPDH2, encoded by *IMPDH2*, in patients with dystonia.^20^ IMP is an important purine intermediate that has a central position at the intersection of the purine de novo synthesis and the salvage pathways (see Fig 1).^34^ The core function of IMPDH2 is to compete with adenylosuccinate synthetase to drive the transformation of IMP toward guanine nucleotides, rather than adenine nucleotides (see Fig 1).^35^ The enzyme is crucial for the survival of organisms, as indicated by the fact that its knockout is lethal in animal models and expressed in all tissues including the developing CNS. IMPDH2ˋs homolog IMPDH1 (encoded by *IMPDH1*) exhibits predominant expression in the retina, where its pathogenic variants can result in an early‐onset progressive form of retinitis pigmentosa.^35^, ^36^ IMPDH2 consists of a 2‐part catalytic domain and an interposed Bateman domain, which is a typical regulatory motif in enzymes capable of allosteric nucleotide binding.^37^ Binding of ATP or guanosine triphosphate (GTP) to different sites within the Bateman domain regulates IMPDH2 structural organization and activity, whereby ATP acts as an activator and GTP as an inhibitor of the enzyme.^38^, ^39^, ^40^, ^41^, ^42^

Work to date has characterized IMPDH2‐associated monogenic disease as a disorder in which dystonia can be either the leading (or sole) symptom or an accompanying feature in the context of more prominent non‐motor neurodevelopmental disturbances (Table 1).^20^, ^21^, ^22^, ^43^ Moreover, *IMPDH2*‐mutated patients have been identified who demonstrated neurodevelopmental syndromes without any signs of dystonia at the time of last examination, highlighting a notable degree of variable expressivity for the condition.^20^, ^22^ Dystonic phenotypes of patients reported to date ranged from infantile‐onset generalized, multifocal, or focal dystonia involving the trunk, limbs, and/or craniocervical districts to childhood‐/adolescence‐onset segmental upper‐body dystonia with or without tremor. Recurrently observed neurodevelopmental features included global developmental delay, axial hypotonia, intellectual disability, seizure activity, and behavioral issues compatible with autism spectrum disorder (for details, see Table 1). Additional variable abnormalities were evident in some affected individuals, such as facial dysmorphia, sleep disturbances, and congenital anomalies (eg, pulmonic stenosis, Table 1). Magnetic resonance imaging (MRI) of the brain was reported for 7 published patients; 5 patients had no evidence of major neuroanatomical changes, whereas 1 patient was found to have polymicrogyria and another 1 cortical heterotopia (Table 1).

**TABLE 1:** Genotypic and phenotypic features of reported patients with *IMPDH2* variants

Variant	Zygosity/inheritance	Reference	(Proposed) mechanism	Functional studies	No. patients	Phenotype (index)	Brain MRI
c.338G>A; p.Gly113Glu	Heterozygous (de novo)	Zech et al^20^	Gain‐of‐function	3D protein modelling,^20^ EM, in vitro activity assay^22^	1	Hypotonia, generalized dystonia, ID	Normal
c.337G>A; p.Gly113Arg	Heterozygous (de novo)	Zech et al^20^	Gain‐of‐function	3D protein modelling,^20^ EM, in vitro activity assay^22^	1	DD, ID, gait instability, autism, sleep disturbance	Normal
c.478_480delTCC p.Ser160del	Heterozygous (de novo)	Zech et al^20^	Gain‐of‐function	3D protein modelling,^20^ EM, in vitro activity assay^22^	1	Hypotonia, focal limb dystonia, DD, ID, autism, facial dysmorphia	Normal
c.619G>C; p.Gly207Arg	Heterozygous (de novo)	Zech et al^20^	Gain‐of‐function	3D protein modelling,^20^ EM, in vitro activity assay^22^	1	DD, ID, seizures, facial dysmorphia	Normal
c.619G>A; p.Gly207Arg	Heterozygous (de novo)	Zechet al^20^	Gain‐of‐function	3D protein modelling, thermal shift assay,^20^ EM, in vitro activity assay^22^	1	DD, ID, epilepsy	Cortical heterotopia
c.729G>C; p.Gln243His	Heterozygous (de novo)	Zech et al^20^	Gain‐of‐function	3D protein modelling, thermal shift assay,^20^ EM, in vitro activity assay^22^	1	Hypotonia, DD, ID, seizures	Polymicrogyria
c.734 T>C; p.Leu245Pro	Heterozygous (de novo)	O'Neill et al^22^	Gain‐of‐function	3D protein modelling, EM, in vitro activity assay^22^	1	Hypotonia, DD, congenital anomalies (pulmonic stenosis, hip dysplasia), cervical dystonia, facial dysmorphia	N/A
c.713A>G; p.Lys238Arg	Heterozygous (de novo)	O'Neill et al^22^	Gain‐of‐function	3D protein modelling, EM, in vitro activity assay^22^	1	Hypotonia, DD, cervical dystonia and limb/trunk posturing (improvement with L‐Dopa therapy), autism	N/A
c.93_96del; p.Tyr32MetfsTer19	Heterozygous (inherited)	Kuukasjärvi et al^21^	Loss‐of‐function	mRNA expression study, protein expression study in fibroblasts, iPSCs, and neurospheres^21^	6 (1 family)	Isolated segmental dystonia, tremor	Normal
c.1296‐1G>T p.?	Heterozygous (inherited)	Lin et al^43^	Loss‐of‐function	Not performed^a^	2 (1 family)	Isolated segmental dystonia	N/A

Dystonic features are highlighted in bold.

Abbreviations: DD = developmental delay; EM = electron microscopy; ID = intellectual disability; iPSCs = induced pluripotent stem cells; MRI = magnetic resonance imaging; N/A = not available.

^a^ Disease‐causing nature of this predicted loss‐of‐function variant remains to be confirmed.

Until now, 9 different heterozygous *IMPDH2* variants with strong evidence of disease causality have been published.^20^, ^21^, ^22^ Experimental data demonstrate that these variants can have gain‐of‐function (n = 8) or loss‐of‐function (n = 1) consequences, highlighting puzzling roles in pathophysiology. The reported alleles comprise 7 missense substitutions, 4 of which mapped to 2 identical amino acid residues, a 1‐amino acid in‐frame deletion, and a frameshift mutation (Table 1). All missense and in‐frame deletion variants appear to cluster around the Bateman domain, predicting an effect on enzyme activity regulation.^22^ Furthermore, the detected missense and in‐frame deletion mutations arose de novo, consistent with the pronounced neurodevelopmental abnormalities presented by the carriers. In contrast, the frameshift alteration, likely not decreasing reproductive fitness, was found to co‐segregate with a milder non‐syndromic dystonia‐tremor phenotype in multi‐generational kindred with multiple affected members.^21^ Two additional rare co‐segregating *IMPDH2* variants were uncovered in a screening of patients with adult‐onset isolated dystonia, but their disease‐causing nature remains to be proven.^43^ Three‐dimensional structure–function modeling suggested that most identified missense and in‐frame deletion variants would disrupt the interplay between the Bateman domain and GTP, causing perturbation of IMPDH2ˋs allosteric regulation, which was confirmed in vitro.^22^ Purified recombinant enzymes containing the patient‐derived variants retained abnormally high levels of activity in the presence of inhibitory GTP concentrations, indicative of a gain‐of‐function effect.^22^ The frameshift *IMPDH2* variant discovered in a large dystonia‐tremor family was shown to exert an opposite effect on IMPDH2 function.^21^ Studies in primary fibroblasts from patients revealed that the variant resulted in degradation of the mutant messenger mRNA and significantly diminished IMPDH2 expression levels compatible with enzyme deficiency, similarly to what has been described for other PMDs.^44^ The lowered amounts of the enzyme were also replicated in induced pluripotent stem cell‐derived neurospheres, indicating IMPDH2 deficiency in the clinically relevant cell type.^21^

## Purine Pool Imbalance and Pathophysiological Implications

The characterization of *IMPDH2* as a new disease gene enhances our knowledge about the relationship between purine metabolism abnormalities and dystonia. In the brain, purine salvage is known to be generally favored over de novo synthesis.^14^, ^45^, ^46^ HGprt deficiency in dystonia‐ and developmental disease‐associated LND triggers increased flux through the de novo synthetic pathway. Overactivity in the de novo pathway is now also supported by the demonstration of gain‐of‐function effects for most identified *IMPDH2* variants.^22^

Moreover, insights from *IMPDH2*‐related disease can help to better understand which type(s) of purine metabolism imbalance(s) is (are) more likely to result in dystonia. HGprt has a principal role in the recycling of nucleotides for both arms of the purine pathway (see Fig 1), rendering it difficult to establish a differential impact on the availability of the guanine versus the adenine pool.^14^, ^47^, ^48^ IMPDH2 is positioned at the key node that determines the fate of IMP toward guanine derivatives.^49^ Therefore, the emerging dominant gain‐of‐function paradigm for *IMPDH2*‐related dystonia could offer clues to better understand disease‐associated molecular drivers and ultimately pathogenesis. Predictably, the altered shunt of purine synthesis toward guanine nucleotides in patients with overactive IMPDH2 may impact negatively on the adenine nucleotide pool (see Fig 1). Considering the multiple essential roles of adenine nucleotides and their derivatives in neurophysiology,^50^, ^51^, ^52^, ^53^ it could well be hypothesized that perturbation of their availability might be even more impactful than a loss of guanine nucleotides, at least in certain neuroanatomical structures causally involved in dystonia. For example, alterations in the availability of adenosine could disturb adenosine A2A signals in dystonia‐relevant cell populations, such as spiny projection neurons and cholinergic interneurons in the basal ganglia.^54^ The hypothesis is supported by the finding that loss‐of‐function variants in ADSL, which is responsible for the conversion of IMP into adenine nucleotides (see Fig 1), and have been implicated in a neurodevelopmental disorder that can manifest with dystonia.^18^ Moreover, the described association of a loss‐of‐function *IMPDH2* variant with dystonia emphasizes a more complex scenario, in which both genetically mediated increases and decreases in guanine and adenine nucleotide levels could be related to disease evolution.^21^ Although future research efforts will need to address the precise relationship between alterations in the purine nucleotide pool and dystonic symptoms in patients, several themes are emerging that link pathomechanistic consequences of purine metabolism perturbance and purine‐pathway enzyme defects to dystonia. We discuss here 3 such relationships, supported by available evidence from LND, *IMPDH2*‐related dystonia and other PMDs, as well as animal or cellular models.

### Neurodevelopmental Abnormalities

**FIGURE 2:**
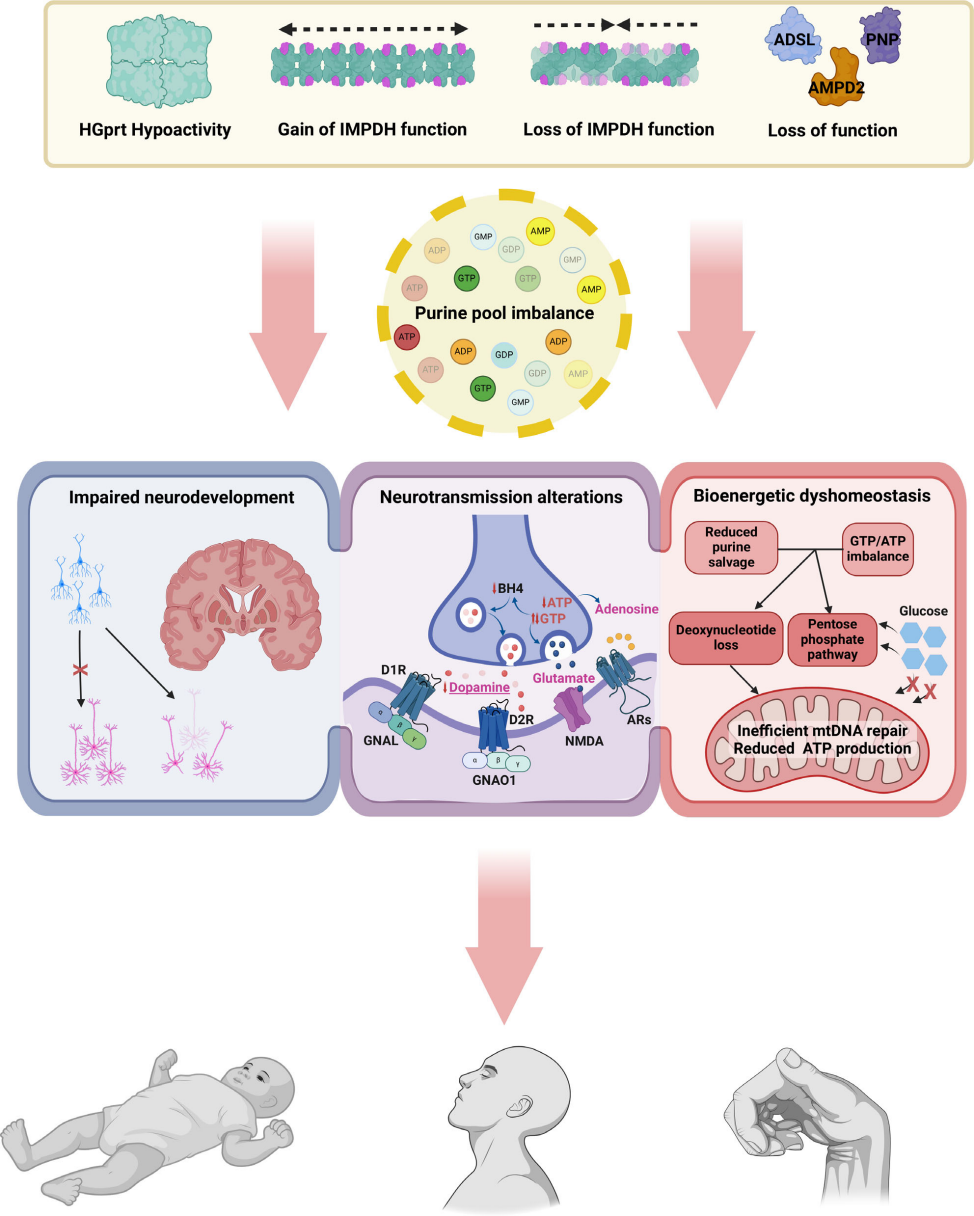
Pathophysiological mechanisms of dystonia in the context of purine metabolism disorders. Pathogenic genetic variants causing alterations in the function of enzymes involved in purine metabolism lead to disruption of purine homeostasis, which is crucial for proper development and maintenance of the organism, particularly the central nervous system. Purine dyshomeostasis is associated with a variety of potentially interconnected pathophysiological disturbances affecting neurodevelopment, neurotransmission, and bioenergetic regulation. When the enzymes HGprt, IMPDH, or, in rarer cases, ADSL, AMPD2, or PNP are involved, the proposed perturbances can result in dystonic phenotypes, putatively because of a more pronounced impairment of basal ganglia development and/or dopaminergic neurotransmission. ADSL = adenylosuccinate lyase; ADP = adenosine diphosphate; AMP = adenosine monophosphate; ARs = adenosine receptors; ATP = adenosine triphosphate; BH4 = tetrahydrobiopterin; D1R = dopamine receptor 1; D2R = dopamine receptor 2; GDP = guanosine diphosphate; GMP = guanosine monophosphate; GNAL = guanine nucleotide‐binding protein, alpha‐activating activity polypeptide, olfactory type; GNAO1 = guanine nucleotide‐binding protein, alpha‐activating activity polypeptide o; GTP = guanosine triphosphate; mtDNA = mitochondrial deoxyribonucleic acid; NMDA = N‐methyl‐D‐aspartate; PNP = purine nucleoside phosphorylase. The figure was created with Biorender.com. [Color figure can be viewed at www.annalsofneurology.org]

Abnormal neurodevelopment is a key feature of many dystonic conditions and a growing number of molecular pathologies converge on neurodevelopmental perturbations in dystonia.^55^, ^56^, ^57^, ^58^ Purines and their derivatives are essentially required for the control of neural cell migration, growth, and differentiation.^59^, ^60^, ^61^ They also have decisive roles in the modulation of synaptic strength during brain development (Fig 2).^62^ Murine models demonstrate that the failure of neural stem and progenitor cells (NSPCs) to synthesize or salvage purine bases has detrimental consequences on CNS proliferation, leading to severe neurodevelopmental trait manifestations.^46^, ^63^

LND is widely recognized as a neurodevelopmental disorder, with a relatively stereotyped evolution of abnormalities that begins with hypotonia and developmental delay, followed by the emergence of abnormal movements, particularly dystonia.^16^ Accompanying neuroanatomical changes are relatively subtle. The brain MRI is usually reported to be clinically normal, but volumetric studies have suggested the entire brain may be small, especially certain regions of the basal ganglia^64^ and deep white matter.^65^ Autopsy studies have failed to reveal any obvious areas of maldevelopment or degeneration.^66^ Moreover, functional studies such as positron emission tomography^67^, ^68^ and several experimental models involving cells^69^ or animals^70^ have raised the possibility that this disorder reflects a problem with neurite extension, showing abnormally underdeveloped dopaminergic nerve terminals and dendrites. Impaired dendritic outgrowth of *HPRT1*‐deficient dopaminergic neurons was shown to result in the failure to develop a sufficiently enriched network of synaptic connections, without affecting their ability to survive.^71^ It has also been hypothesized that effective purine salvage is necessary for the formation of midbrain dopaminergic cells during critical developmental stages.^72^, ^73^ Furthermore, the arborization process of dopaminergic terminals in the basal ganglia during early development is believed to be highly demanding in terms of nucleotide consumption.^74^ These observations highlight an important role of the HGprt‐related purine salvage pathway in the development of dopaminergic neurons.^75^ However, this type of anatomical defect is challenging to establish in human brains, so the actual underlying anatomical defect (if any) remains to be proven. Most attention has focused on a dopamine neuron projection from the midbrain to the basal ganglia,^76^ although it seems likely that many brain regions may be affected by a similar fundamental problem.^77^ Furthermore, recent work by Mizukoshi and colleagues^46^ demonstrated that the purine salvage pathway is highly active during the differentiation of NSPCs into granule cells in the cerebellum, suggesting additional perturbations of cerebellar development in LND. Currently, there is no clear molecular explanation for the observed abnormalities of dopaminergic cell development in patients with *HPRT1* mutations. The purine sensor RAS homolog enriched in brain (RHEB) has been studied for its role as an activator of mTORC1, a canonical protein kinase linked to cell survival, proliferation, and differentiation, which may also be involved in the development of dopaminergic cells in the midbrain. Reduced expression of RHEB because of HGprt‐related purine depletion might lead to incomplete activation of mTORC1 and a consequent lack of cellular differentiation.^72^ Results of other studies suggested that the development of dopaminergic neurons might be directly affected by alterations in adenosine‐dependent signaling, highlighting the roles of the A2A and A3 adenosine receptors.^13^, ^78^ Finally, it is now understood that the expression levels of a number of specific neurodevelopmental genes can be altered in the context of *HPRT1*‐related purine metabolism perturbance, including genes that are vital for dopaminergic neuron and/or striatal development such as *FOXG1*, *NR4A2*, and *BCL11B*.^79^ These 3 genes have also been linked to autosomal dominant diseases that can manifest with prominent dystonia.^80^, ^81^, ^82^ In HGprt‐deficient striatal cells, *BCL11B* downregulation was found to be coupled to dysregulated expression of DARPP‐32, a central integrator of dopaminergic neurotransmission with links to multiple neuronal signals, associating purinergic system dysfunction with a wide range of defects in neurophysiological connectivity.^79^

In patients with *IMPDH2* mutations, neurodevelopmental disturbances were described as the most consistent clinical abnormality.^20^ In 2 subjects with gain‐of‐function missense variants, signs of abnormal development of the brain before birth were reported in the forms of polymicrogyria or cortical heterotopia (Table 1).^20^ Important developmental roles for IMPDH2 are also evident from a published conditional *Impdh2* mutant mouse showing defects of cellular proliferation during neural crest development, compatible with an insufficient guanine‐nucleotide supply of specialized cell populations affected by the enzyme depletion.^83^ Although the precise mechanisms of neurodevelopmental dysfunction in *IMPDH2*‐related dystonia remain largely unexplored, preliminary evidence from Toyoda and colleagues indicates that IMPDH2 filaments are enriched during development in the distal ends and branching sites of astrocytes.^84^ Glial dysfunction is an increasingly appreciated contributor to dystonia pathogenesis, highlighting studies into the role of abnormal astrocytogenesis in *IMPDH2*‐related dystonia as a promising future research topic.^85^ IMPDH2 has been more extensively examined for its implication in neuronal health in the adult brain. Data from different studies show that proper IMPDH activity enforces neuroprotective mechanisms, which might prevent neurodegeneration.^86^, ^87^ Woulfe and colleagues^88^ raised the question of whether the physiological mobility of higher‐order organizations of IMPDH as filaments within the cell could be affected by pathogenic *IMPDH2* variants, especially those that cause IMPDH to be constitutively in an altered conformation. Intracellular structural changes of IMPDH filaments such as crystallized nuclear inclusions were observed in postmortem samples from substantia nigra dopaminergic neurons, potentially related to neurodegenerative consequences.^88^ As such, *IMPDH2*‐related disease may serve as a useful novel model to study neurodevelopmental versus neurodegenerative mechanisms in dystonia.

Ultimately, findings from other human monogenic diseases underline the importance of purine metabolism for neurotypical development.^89^ A number of additional PMDs, disrupting the de novo synthesis or the salvage pathways, have been associated with clinical syndromes characterized by neurodevelopmental delay, intellectual disability, movement disorders, and congenital brain malformations (see Fig 1).^59^, ^90^, ^91^, ^92^ Although in some cases the neurodevelopmental impairment is thought to be the consequence of toxic substrate accumulation, such as in ADSL deficiency and 5‐amino‐4‐imidazolecarboxamide‐ribosiduria because of ATIC deficiency,^93^ other conditions including Arts syndrome (phosphoribosylpyrophosphate synthase deficiency) and severe combined immunodeficiency because of adenosine deaminase deficiency show evidence of more subtle alterations of CNS developmental processes.^92^, ^94^

### Neurotransmitter Imbalance

Defects in neurotransmitter synthesis and synaptic transmission represent established key factors in the pathogenesis of dystonia.^95^, ^96^ Mounting evidence indicates that purines and their derivatives, together with specific receptors and other interacting molecules, profoundly influence the physiological integrity of neurotransmission within the CNS.^5^ Purines serve as endogenous ligands for the activation of a wide array of purinergic signaling pathways, regulating multiple downstream secondary messengers such as Ca^2+^ and IP3 (see Fig 2).^97^, ^98^, ^99^ Disruption of Ca^2+^ homeostasis has been associated with the pathogenesis of diverse genetic and acquired forms of dystonia,^100^, ^101^, ^102^ whereas IP3 receptor defects were shown to lead to cerebellar Purkinje cell firing alterations and dystonic movements in mice.^103^ More broadly, purinergic neurotransmission has been characterized as an essential modulator of proliferation, differentiation, and migration of NSPCs, controlling neurodevelopment and the maintenance and regeneration of neural tissue in the adult brain.^13^, ^46^, ^86^, ^104^

Several studies investigated the consequences of imbalanced quantities of purine nucleotides, caused by HGprt deficiency, in the context of neurotransmitter dyshomeostasis. Diminished activity of a number of dopaminergic functions is a main molecular characteristic of LND and likely to be responsible for associated symptoms including dystonia.^70^ Basal ganglia of patients with LND were found to contain reduced amounts of dopamine (70–90% decrease) and this finding was replicated in different animal models.^66^, ^105^, ^106^ In addition to developmental abnormalities directly affecting the functional state of dopaminergic neurons, suboptimal levels of tetrahydrobiopterin (BH4), which is produced from the precursor GTP, were discussed to aggravate dopamine defects in LND (see Fig 2).^107^ BH4 is the essential cofactor required for the conversion of tyrosine to dopamine. Purine metabolism abnormalities in LND involve more complex changes in neurotransmitter regulation, which include specific alterations in the levels of receptors, transporters, and dopamine cotransmitters. It has been reported that the expression of D1 and D2 dopamine receptors in the putamen and caudate nucleus of patients with LND is increased, whereas presynaptic enzymes and recycling proteins involved in dopamine metabolism, such as tyrosine hydroxylase, DOPA decarboxylase, dopamine transporter 1, and vesicular monoamine transporter, seem to be consistently downregulated.^70^ Moreover, it was observed that loss of dopamine in the nigrostriatal pathway is accompanied by relative preserved synaptic action of the neurotransmitters norepinephrine, serotonin, and GABA.^70^, ^108^ Histamine is increased in animal models, possibly to compensate for reduced availability of dopamine by facilitating its release.^109^ Glutamatergic neurotransmission, a known component in the neurophysiology of dystonia, is also considered to be influenced by purine metabolism dysfunction.^71^ Guanosine is an established modulator of L‐glutamate activity, promoting its reuptake by astrocytes.^110^ A reduced pool of guanosine resulting from impaired rescue of guanine is predicted to lead to deregulation of glutamatergic signaling (see Fig 2). This might have negative effects on neuron and astrocyte survival, as elevated levels of L‐glutamate are excitotoxic and can cause cell death.^111^ Finally, it has been speculated that altered purine levels could impact on the functionality of certain neurotransmitter receptors, and this may provide another mechanistic link to the manifestation of dystonia in PMDs.^89^ G protein‐coupled receptors (GPCR) depend heavily on the availability of guanine nucleotides, because these transmembrane complexes require GTP and guanosine diphosphate to perform molecular switches in activity (see Fig 2).^112^ GPCR are of considerable importance for postsynaptic signaling in striatal neurons, where they act as dopamine receptors including D2 receptors, which have been linked to autosomal dominant early‐onset dystonia. Different subtypes of the GPCR‐associated, GTP‐hydrolyzing G proteins such as GNAL, GNAO1, and GNB1 play essential physiological roles in the regulation of neurons that mediate movement control and were found to be mutated in patients with isolated and complex dystonic syndromes.^113^, ^114^, ^115^

Although it has not been directly shown that dystonia‐linked *IMPDH2* mutations result in purine pool imbalance, findings from in vitro experiments demonstrating a gain of IMPDH2 function predict that the relative levels of adenine nucleotides would be disrupted by an increase in the ratio of GMP to AMP synthesis (see Figs 1 and 2).^22^ Secondary effects in patients could be reductions in the functional concentrations of adenosine and/or ATP (Figure 2). The adenosine system exhibits an ubiquitous distribution in the brain with high abundance in striatopallidal neurons and functions as an upstream regulator of excitatory and inhibitory neurotransmission. It was shown that impaired regulation of fine‐tuned adenosine levels can give rise to aberrant dopaminergic neurotransmission and changes in synaptic plasticity, processes that have been identified as recurrent pathophysiological themes in dystonia.^116^, ^117^ Similarly, ATP is a constitutive cotransmitter that exerts important neuromodulatory roles in concert with dopamine, glutamate, GABA, and acetylcholine, exhibiting widespread presence in synaptic networks involving neurons and glial cells.^53^ Experimental evidence indicates that ATP‐mediated signaling participates in neurodevelopment and maintenance of normal adult brain function, sensory system integration, as well as microglial responses to inflammatory challenges.^118^, ^119^

### Bioenergetic and Mitochondrial Dyshomeostasis

Another pathological process that is involved in the genesis of dystonic movements is cellular energy deficiency. Based on clinico‐genetic observations in patients, in vitro studies, and animal models, pathways required for energy production and transfer in the brain are often compromised in dystonic syndromes.^120^, ^121^, ^122^ Both purine de novo synthesis and purine salvage are closely connected to cellular energetic states and mitochondrial function.^123^, ^124^ Purines and their downstream signals are crucial to sustain the orchestration of energy homeostasis that is required for neuronal activity and associated astrocytic responses.^5^, ^125^

In situations where the de novo and salvage pathways become discoordinated, cells may experience higher energy demands to maintain purine pools that are essential to meet the requirements for normal function.^126^ Such shifts in pathway utilization would be the expected outcome of several purine‐pathway enzyme defects resulting in PMDs, including LND and, potentially, *IMPDH2*‐related dystonia caused by gain‐of‐function mutations (see Fig 2). Mechanistically, imbalances driving purine de novo synthesis to become more active would lead to an enhanced activation of biochemical pathways that depend on glucose concentrations. Direct experimental confirmation for disturbances in glucose metabolism as a result of greater reliance on the de novo synthetic pathway in PMDs comes from studies of neural cells derived from patients with LND. Bell and colleagues^72^ performed real‐time glucose tracing to reveal that HGprt deficiency causes an increased shunt of glucose to the pentose phosphate pathway, which is required to maximize the efficiency of purine de novo synthesis. This, in turn, resulted in reduced availability of glucose for glycolysis and oxidative phosphorylation, culminating in a negative impact on ATP production. When assessing cell type‐specific consequences of the glucose shunting, the authors observed that energy defects were most evident in dopamine neurons, whereas some cortical cells were less severely affected, highlighting pronounced vulnerability of a main dystonia‐relevant system.^72^ Similar alterations in energetic metabolism could be hypothesized for gain of IMPDH2 function, although the associated disease pathophysiology at the cellular level still remains largely elusive.

Energy states of brain regions required for movement control can be further impaired by oxidative stress and mitochondrial dysfunction. In LND‐affected individuals, certain cells may suffer from significantly increased oxidative stress, because failure of the salvage for hypoxanthine leads to increased substrate availability for xanthine oxidase, which catalyzes a reaction generating reactive oxygen species.^127^ In a knockout mouse model, purine salvage impairment was found to be associated with increased markers of oxidative stress such as glutathione oxidation and lipid peroxidation, compatible with a contribution of oxidative injury in the pathogenesis of purine metabolism perturbance‐related disease phenotypes.^128^ Furthermore, the intersection between purine metabolism and mitochondria is increasingly appreciated, which may shed new light on the evolution of PMDs with dystonic features.^129^, ^130^ For their proper function, mitochondria depend on a continuous supply with purine‐derived deoxynucleotides.^131^ In several monogenic disorders leading to disruption of synthesis or transport of mitochondrially required deoxynucleotides, insufficient mitochondrial DNA repair and low replication fidelity were observed, resulting in mitochondrial depletion and neurological impairment.^132^, ^133^ Conversely, mitochondrial defects are likely to aggravate dysregulated ATP metabolism (see Figs 1 and 2). Moreover, the ultimate products of mitochondrial metabolism such as ATP are important cofactors for enzymes in the purine de novo synthetic pathway.^134^ A direct link between purinergic system abnormalities and mitochondrial function impairment has been demonstrated in a very recent study by Vinokurov and colleagues,^129^ showing defective mitochondrial respiration, altered mitochondrial membrane potential, and increased reactive oxygen species (ROS) levels in *HPRT1*‐mutated murine midbrain neurons. Excessive production of ROS can interfere with dopamine metabolism and CNS redox homeostasis, impairing neural cell viability and anti‐inflammatory reactivity.^135^

## Dystonia Molecular Pathways at the Intersection of Purine Metabolism Perturbance: Potential for Targeted Treatment?

Pathogenic mechanisms in dystonia are extremely heterogeneous.^55^ Nevertheless, the continuous progress in knowledge derived from studies of monogenic dystonia has led us to hypothesize that products encoded by dystonia‐associated genes may play a role in shared molecular pathways.^136^ It is still unclear to what extent such relationships could be the consequence of immediate interaction or an overarching mechanistic network. Linking dystonia‐related gene products may be arbitrary, because concepts of interconnected networks are often based on too simplistic functional annotation, which might not be fully reflective of true biological scenarios in the diseased brain. Despite such a cautionary note, convincing evidence demonstrates that unifying mechanisms linking multiple pathophysiological cascades in dystonia are starting to emerge.^136^

Here, we reviewed evidence to reinforce the proposition that purine metabolism perturbance is likely to be interconnected with major dystonia‐relevant pathomechanisms. These mechanisms include abnormal neurodevelopment, neurotransmitter perturbations with a prominent role of dopaminergic deficits, as well as energy and mitochondrial impairment (see Fig 2). Important similarities are evident between the molecular changes observed in studies of PMDs, especially those caused by mutations in *HPRT1* and *IMPDH2*, and key pathological themes in dystonia. Although much remains to be clarified, for example, how *IMPDH2* gain‐ and loss‐of‐function mutations confer pathogenicity in the cellular context, it is well conceivable that intersections of different biological dysfunctions observed in PMDs and other monogenic dystonias may create a defined set of key multi‐pathway loops that could be of particular etiological relevance.

**FIGURE 3:**
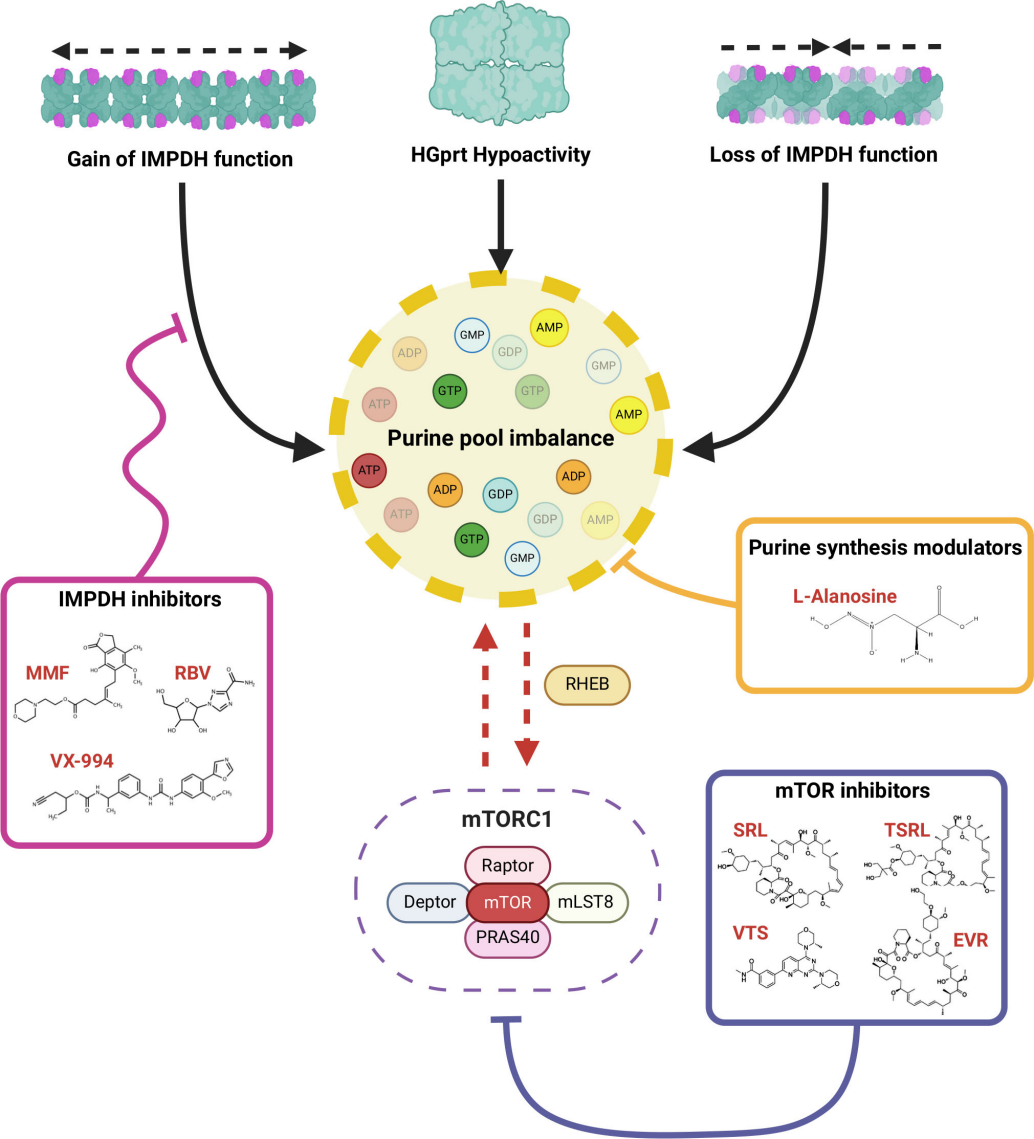
Potential therapeutic strategies for purine metabolism disorders with dystonia. Because of its central role in various pathophysiological processes, such as cancer and immune disorders, purine metabolism is regarded as an attractive target for pharmacological treatment. Repurposing of drugs targeted against purine metabolism defects could represent a new avenue for therapeutic intervention in dystonia and neurodevelopmental disease. Dystonia caused by *IMPDH2* gain‐of‐function variants may be amenable to treatment with IMPDH‐inhibitors such as mycophenolate mofetil or ribavirin. The effect of the ADSL inhibitor L‐alanosine on purine homeostasis could also be explored. The influence of purine levels on ubiquitous metabolic and growth regulators such as mTORC1 highlights an additional possible role for inhibitors of this pathway. ADP = adenosine diphosphate; AMP = adenosine monophosphate; ATP = adenosine triphosphate; EVR = Everolimus; GDP = guanosine diphosphate; GMP = guanosine monophosphate; GTP = guanosine triphosphate; HGprt = hypoxanthin‐guanine phosphoribosyltransferase; IMPDH = inosine monophosphate dehydrogenase; mLST8 = mammalian lethal with SEC13 protein‐8; MMF = Mycophenolate mofetil; mTOR(C) = mammalian target of rapamycine (complex); PRAS40 = proline‐rich akt substrate, 40‐kd; RBV = Ribavirin; RHEB = RAS homolog enriched in brain; SRL = Sirolimus; TSRL = Temsirolimus; VTS = Vistusertib. The figure was created with Biorender.com. [Color figure can be viewed at www.annalsofneurology.org]

Evolving insights into such pathway links can foster the development of etiology‐directed therapies (Fig 3). Enhanced understanding of commonly impaired mechanistic nodes in dystonia could provide a stimulus for treatment discovery and establishment of drug repurposing through an increased number of individuals that would be eligible for a targeted trial. Importantly, the discovery of *IMPDH2* mutations as a new cause of dystonia may offer fresh innovative avenues for therapeutic interventions that target a central disease‐associated node (Figs 1, 2, 3). Because of its essential functions in development and proliferation, IMPDH is an established target for immunosuppressive and anticancer medications.^137^, ^138^, ^139^ These existing compounds could be effectively repurposed for the modulation of patient‐specific *IMPDH2* gain‐of‐function mutations given that their mode of action is often IMPDH inhibition. Available drug opportunities to inhibit IMPDH include, for instance, the immunosuppressant mycophenolate mofetil and the virostatic agent ribavirin.^140^, ^141^ Testing potential beneficial effects of purine de novo synthesis inhibition (eg, with L‐alanosine) could be another potential strategy that may deserve consideration.^142^ Moreover, the observation that imbalances in purine nucleotide availability affect mTORC1 activity suggests that pharmacological manipulation of this complex might represent another possible therapeutic approach (see Fig 3).^72^, ^143^

A key interest in the field of dystonia is to determine translatability of findings from rare monogenic subtypes to a broader group of affected individuals.^55^, ^136^ In this regard, further research is needed to explore whether IMPDH‐ and/or other purine synthetic enzyme‐targeted treatments could be introduced as efficient pharmacological approaches that aim to tackle one or more converging pathogenic pathways in dystonia.

## Concluding Remark

Accumulating evidence indicates that purine metabolism enzyme defects contribute to the pathogenesis of dystonic diseases. We have summarized key findings that indicate mechanistic connections between purinergic system dysfunction and dystonia at the clinical, developmental, functional, and molecular levels. Although some of the discussed relationships have not yet been tested experimentally, we hope that our views will inspire investigators to conduct more in‐depth studies into the mechanisms of action of purine perturbance in dystonia. Despite profound gaps in our knowledge about the precise pathophysiology, drugs targeting purine metabolism may be of potential interest for future experimental therapeutic applications in neurodevelopmental disorders and dystonia.

## Author Contributions

U.S., A.G.O.N., J.M.K., H.A.J., and M.Z. contributed to the conception and design of the manuscript; U.S., A.G.O.N., J.M.K., H.A.J., and M.Z. contributed to the interpretation of studies included in the manuscript; U.S., H.A.J., and M.Z. contributed to drafting the text and preparing the figures.

## Potential Conflicts of Interest

Nothing to report.

## Data Availability

Not applicable.
